# Supplementary material for: Heavy-to-light electron transition enabling real-time spectra detection of charged particles by a biocompatible semiconductor
Source: Nat Commun. 2024 Feb 6;15:1115. doi: 10.1038/s41467-024-45089-2 (PMC10847108; doi:10.1038/s41467-024-45089-2)
Supplement: Supplementary file 1 — Supplementary Information [file 41467_2024_45089_MOESM1_ESM.pdf]

Supplementary Information of

Heavy-to-Light Electron Transition Enabling Real-Time Spectra Detection of Charged Particles by A Biocompatible Semiconductor

Dou Zhao[1, 2], Ruiling Gao[3, 4], Wei Cheng[5], Mengyao Wen[1], Xinlei Zhang[6], Tomoyuki Yokota[2], Paul Sellin[7], Shengyuan A. Yang[4], Li Shang[1], Chongjian Zhou[1], Takao Someya[2]*, Wanqi Jie[1]*, Yadong Xu[1]*

[1]State Key Laboratory of Solidification Processing, Northwestern Polytechnical University, Xi'an, Shaanxi, 710072, China.

[2]Department of Electrical Engineering and Information Systems, The University of Tokyo, Tokyo, 113-8656, Japan.

[3]International Center of Quantum and Molecular Structures, Shanghai University, Shanghai, 200444, China.

[4]Research Laboratory for Quantum Materials, Singapore University of Technology and Design, Singapore, 487372, Singapore.

[5]Department of Nuclear Science and Engineering, Nanjing University of Aeronautics and Astronautics, Nanjing, 211106, China.

[6]School of Physics and Information Technology, Shaanxi Normal University, Xi'an, Shaanxi, 710119, China.

[7]Department of Physics, University of Surrey, Guildford, Surry, GU2 7XH, UK.

*E-mail: someya@ee.u-tokyo.ac.jp (T. Someya); jwq@nwpu.edu.cn (W. Jie); xyd220@nwpu.edu.cn (Y. Xu).

Table of Contents

Fig. S1 ... 3
Fig. S2 ... 4
Fig. S3 ... 5
Fig. S4 ... 6
Fig. S5 ... 7
Fig. S6 ... 8
Fig. S7 ... 9
Fig. S8 ... 10
Fig. S9 ... 11
Fig. S10 ... 12
Fig. S11 ... 13
Fig. S12 ... 14
Fig. S13 ... 15
Fig. S14 ... 16
SI 1 ... 17
Fig. S15 ... 18
Table. S1 ... 19
Fig. S16 ... 20
SI 2 ... 21
Fig. S17 ... 22
Fig. S18 ... 23
Fig. S19 ... 24
SI 3 ... 25
Fig. S20 ... 25
Fig. S21 ... 26
SI 4 ... 27
Fig. S22 ... 27
SI 5 ... 28
Fig. S23 ... 30
Fig. S24 ... 30
Fig. S25 ... 31
SI 6 ... 32
Fig. S26 ... 33
Fig. S27 ... 33
Fig. S28 ... 34
Table. S2 ... 35
Fig. S29 ... 36
References ... 37

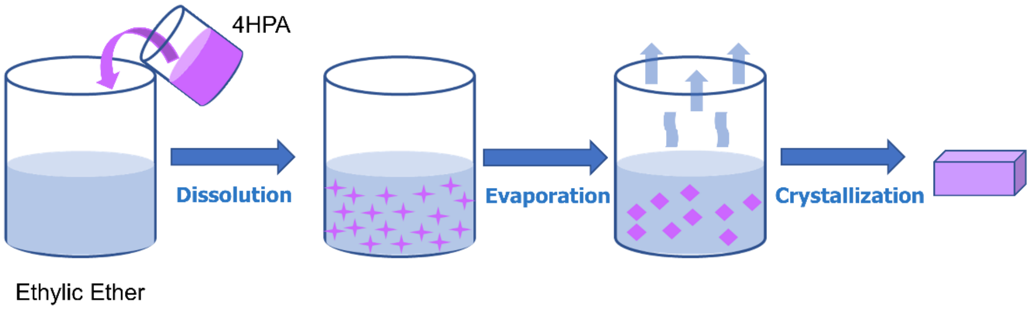

Fig. S1 Schematic diagram of solvent evaporation method for 4HPA single crystals growth.

By carefully controlling the nuclear rate by solution supersaturation, the isolated millimetre-size 4HPA single crystals can be obtained. The low temperature (0~5 °C) and controlled solvent evaporation rate were utilized for 4HPA growth, detailed control method has been reported in our previous paper for 4HCB organic single crystals[1].

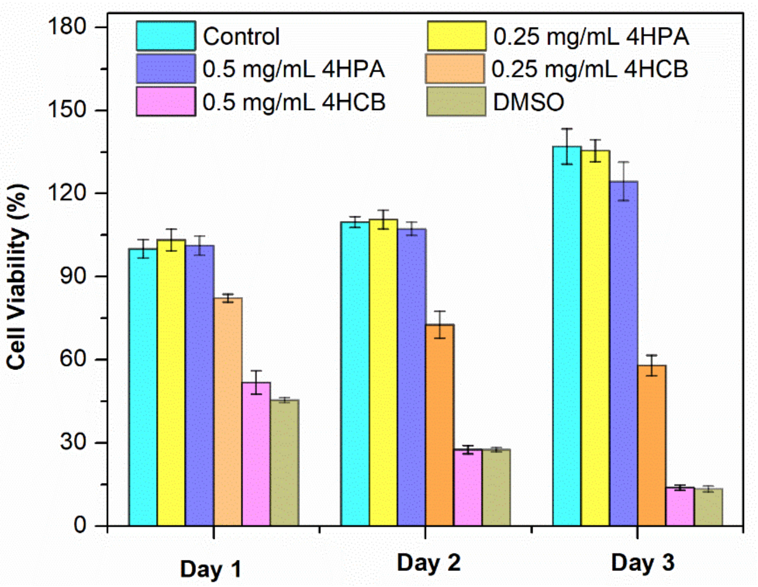

Fig. S2 Cell viability of different incubation groups after 1, 2, and 3 days of incubation. 4HPA with concentration of 0.5 mg/ml show very good biocompatibility even after 3 days of incubation, while the incubation group with 0.25 mg/ml 4HCB show significant cell viability decrease at day 1.

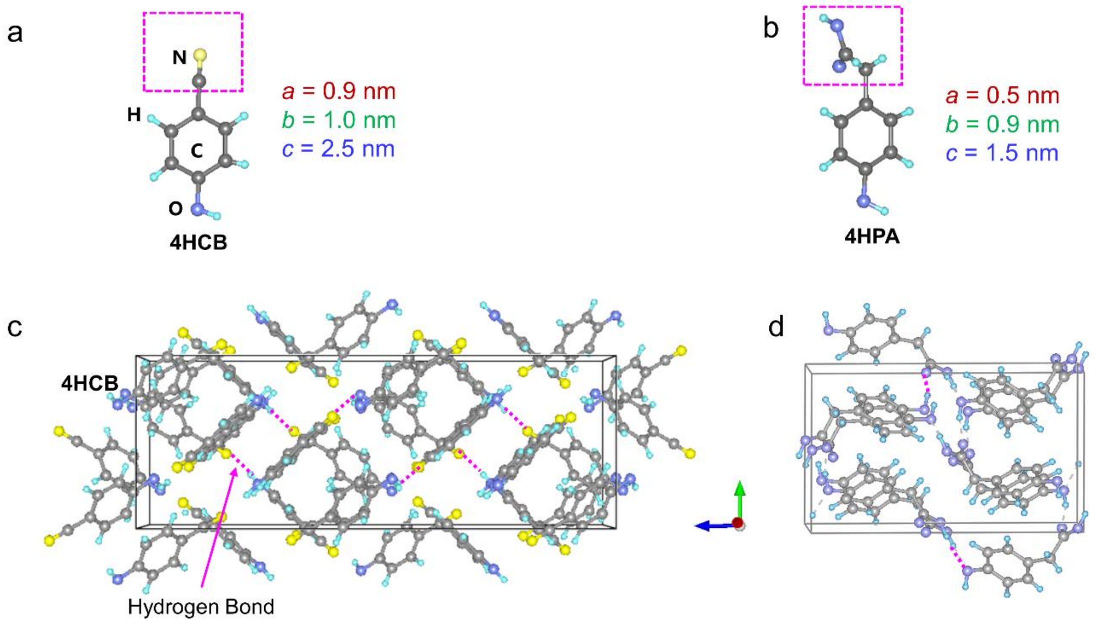

Fig. S3 Molecular structures of 4HCB and 4HPA. Single molecular structures of (a) 4HCB and (b) 4HPA, molecular packing of (c) 4HCB and (d) 4HPA. 4HPA and 4HCB molecules only have one different function group, the -CN group in 4HCB while -CH_2COOH group in 4HPA. Compared with -CN group, -CH_2COOH group results in better biocompatibility of 4HPA (Fig. S3(a-b)). In addition, the difference of the functional group in 4HCB and 4HPA molecules also induces similar packing type but different periodical properties in single crystals (Fig. S3(c-d)). For example, 4HPA has similar quasi-two-dimensional crystal structures as 4HCB, with intermolecular bonds along the a and b axes mainly rely on the $\pi - \pi$ bonds while that of the c axis is hydrogen bonds. However, compared with 4HCB, the distance (unit cell along main crystal axes: a = 0.5 nm, b = 0.9 nm, c = 1.5 nm) between two neighbouring molecular with $\pi - \pi$ bonds and hydrogen bonds are smaller than 4HCB (unit cell along main crystal axes: a = 0.9 nm, b = 1.0 nm, c = 2.5 nm). This indicates that the charge transport properties of 4HPA is better than 4HCB due to higher-degree of $\pi - \pi$ overlaps of neighbouring molecules.

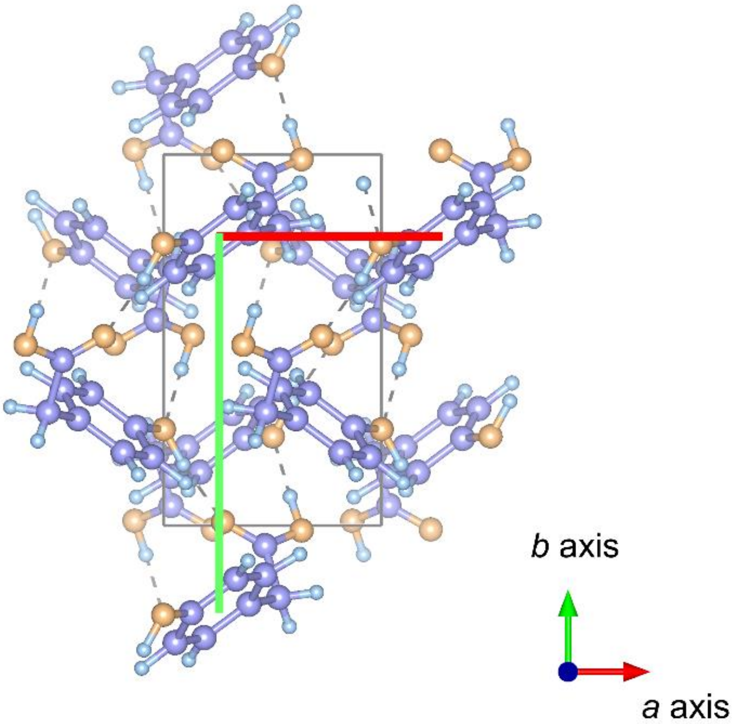

Fig. S4 Projection of 4HPA single crystal structures along the *c* axis. This figure shows that the distance between two overlapped benzene rings along the *a* axis (red line) is 0.5 nm, along the *b* axis (green line) is 0.9 nm.

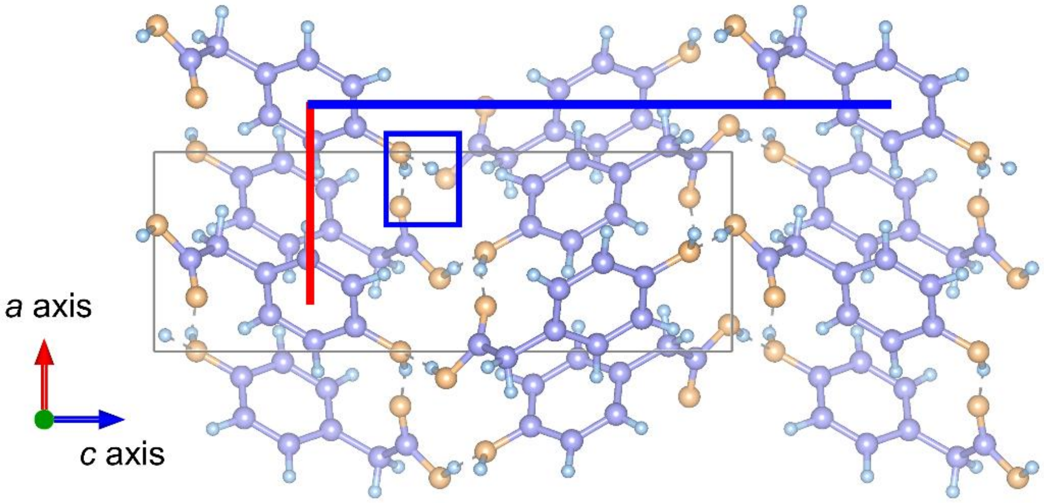

Fig. S5 Projection of 4HPA single crystal structures along the *b* axis. This figure shows that the distance between two overlapped benzene rings along the *a* axis (red line) is 0.5 nm, along the *c* axis (blue line) is 1.5 nm. Along the *c* axis, the role of hydrogen bonds (blue box) is significant.

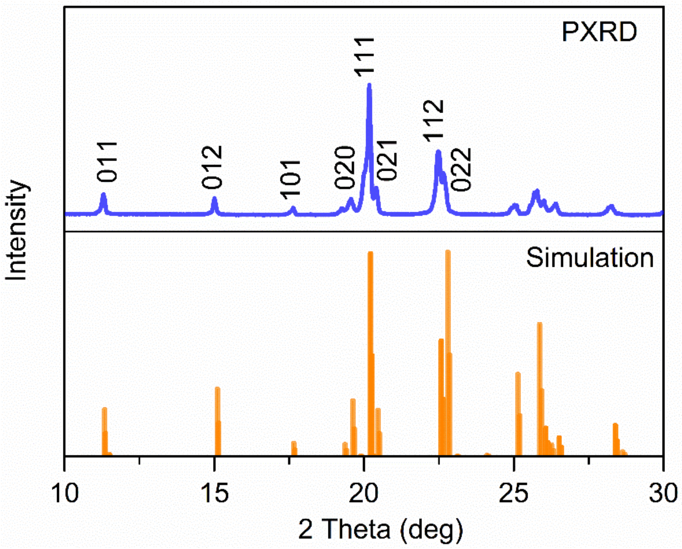

Fig. S6 Powder XRD pattern of 4HPA crystals. The measured powder XRD pattern of as-grown 4HPA crystals is consistent well with the simulated result by VESTA using CIF file (Identifier: QAPBAL, Deposition Number: 274674) from the CCDC database.

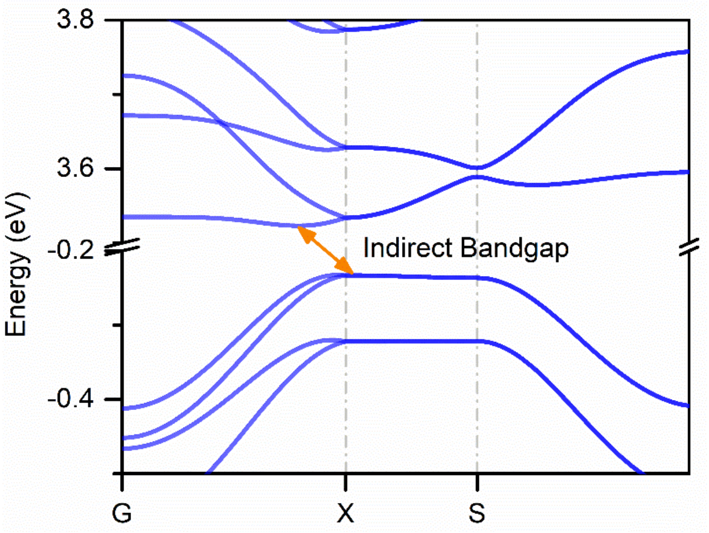

Fig. S7 Enlarged band structure of 4HPA single crystals. This figure indicates that 4HPA has the indirect bandgap.

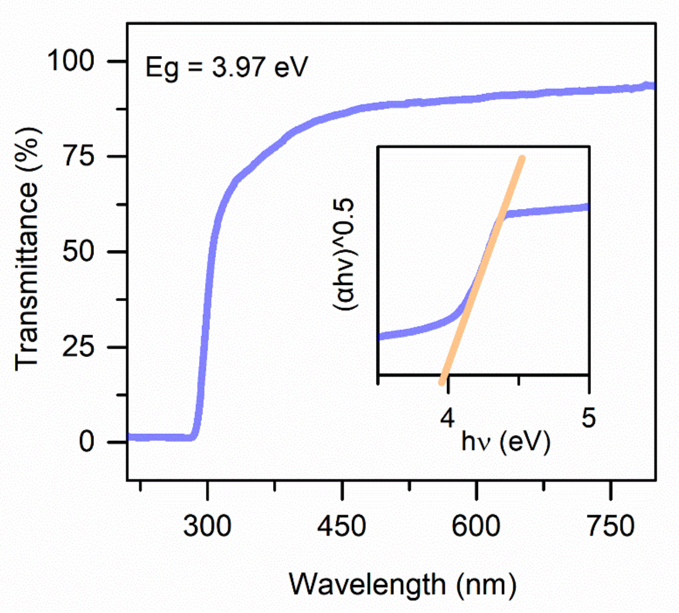

Fig. S8 UV-Vis transmittance spectrum of 4HPA single crystals with insertion is Tauc plot fitting for band gap. For 4HPA single crystal with indirect bandgap, the band gap is calculated using the equation $(\alpha h v)^{1/2} = B\ (hv - E_g)$, α is the absorption coefficient, h is Planck's constant, v is the frequency, B is the constant, E_g is the bandgap[2].

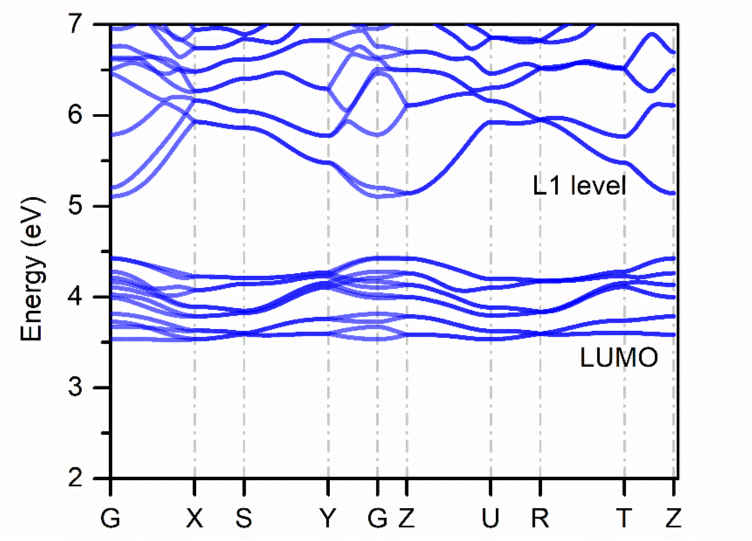

Fig. S9 Enlarged band structure of 4HPA single crystals. This figure indicates that LUMO level of 4HPA is flatter when compared with the L1 level.

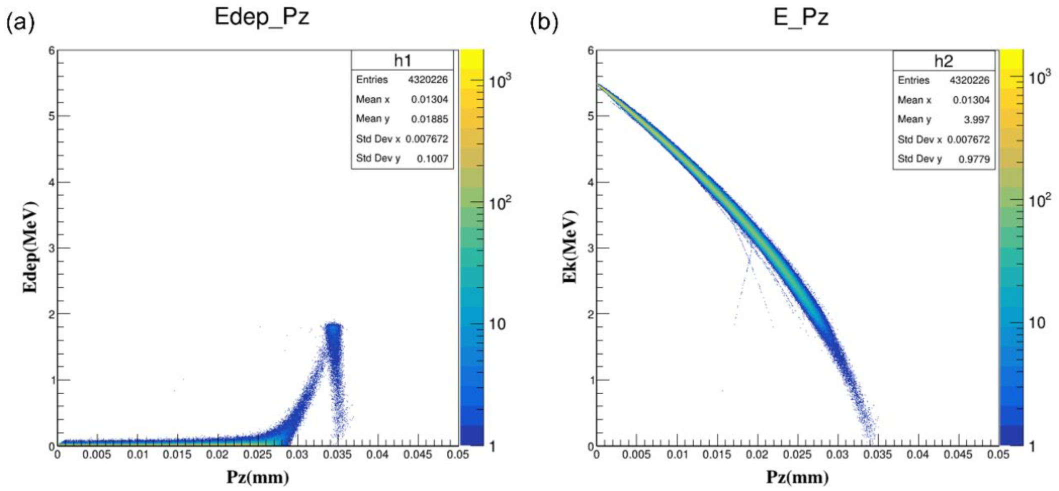

Fig. S10 Energy deposition of 5.49 MeV α particles. (a) Energy deposition in 4HPA detectors changes with sample thickness, (b) the energy of incident α particles changes with penetration depth.

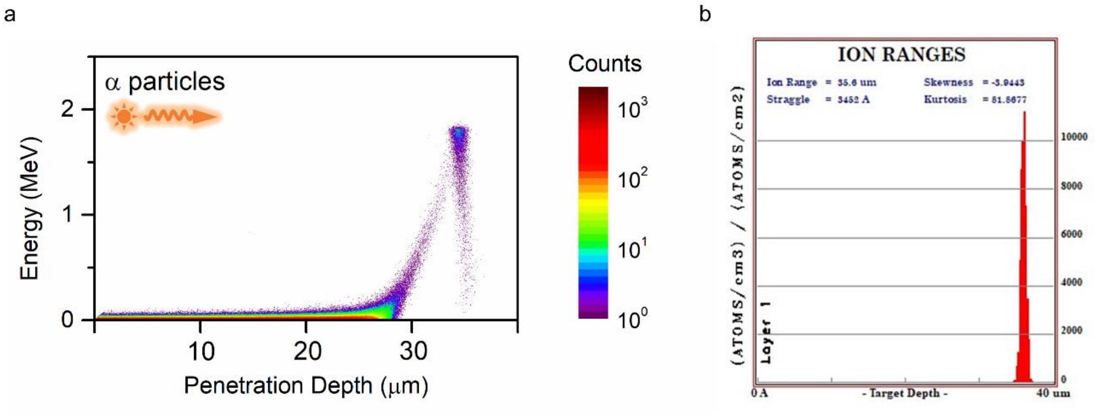

Fig. S11 Energy deposition of 5.49 MeV α particles in 4HPA detectors simulated by (a) Geant4 software, and (b) SRIM software.

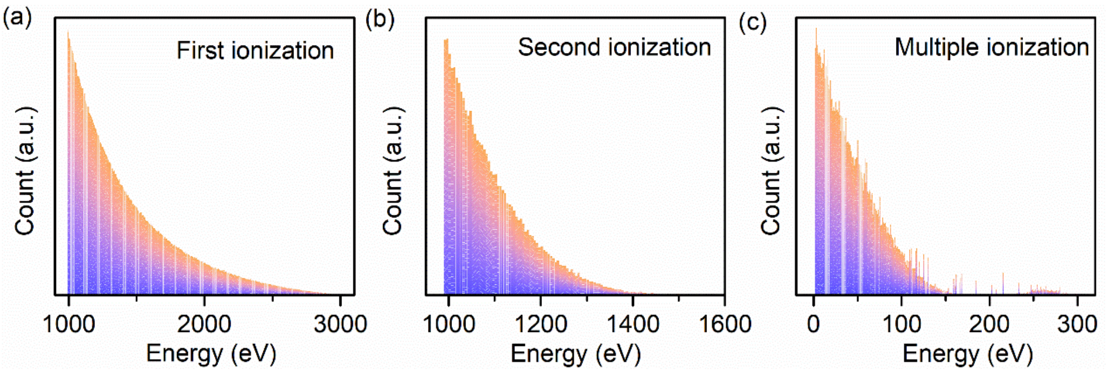

Fig. S12 Energy distribution spectra of electrons ionized by 5.49 MeV α particles. (a) alpha particles ionized electrons, (b) second ionized electrons, (c) multiple ionized electrons.

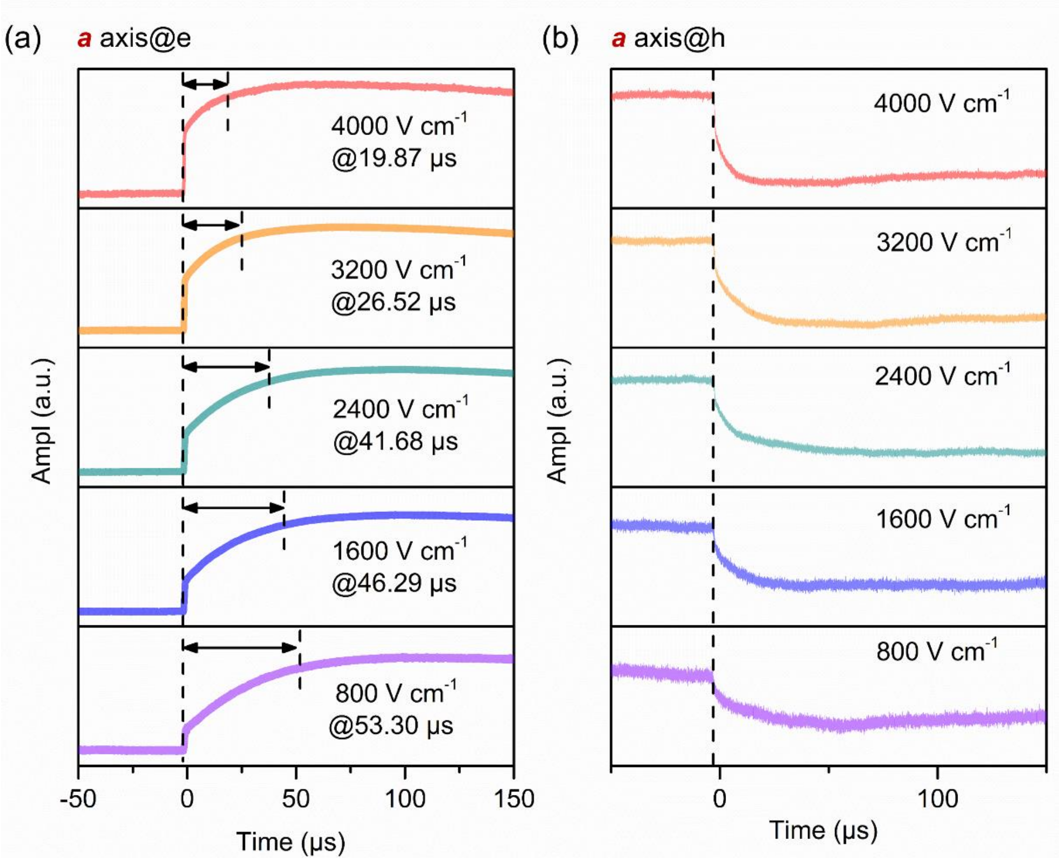

Fig. S13 Charge carrier transport behaviour in 4HPA single crystal along the *a* axis. (a) Electron-only charge rising pulses (averaged by 100 single electron-only pulses) under a series of electric fields, (b) hole-only charge drift time under a series of electric fields.

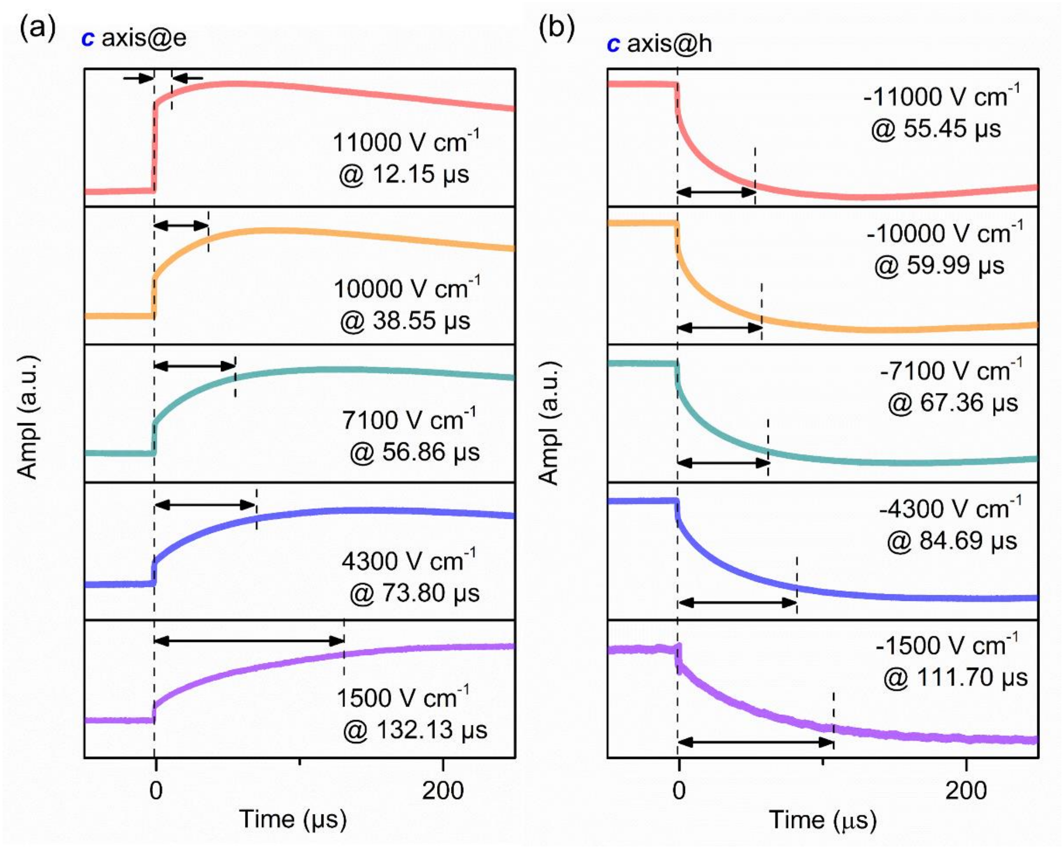

Fig. S14 Charge carrier transport behaviour in 4HPA single crystal along the *c* axis. (a) Electron-only charge rising pulses (averaged by 100 single electron-only pulses) under a series of electric fields, (b) hole-only charge drift time under a series of electric fields.

SI 1 TOF theory about the amplitude of the rising pulse.

According to TOF theory, the amplitude of the rising pulse $q(t)$ can be expressed[3],

$$q(t) = q_0(1 - \exp(-\frac{\mu E t}{\omega})) \qquad \text{(S1)}$$

$$q_0 = \frac{n_0 e \omega}{d} \qquad \text{(S2)}$$

where n_0 is the initial number of released electron-hole pairs by incident α particles, e is the elementary charge, $\omega = \mu E T$, is average drift distance corresponding to the T that is average drift time of conduction electrons or holes before being trapped, μ is carrier mobility, E is electric field, d is distance between two electrodes. Therefore, the saturated amplitude is approximately equal to q_0, and thus is positive to μT (when E, d and n_0 are determined).

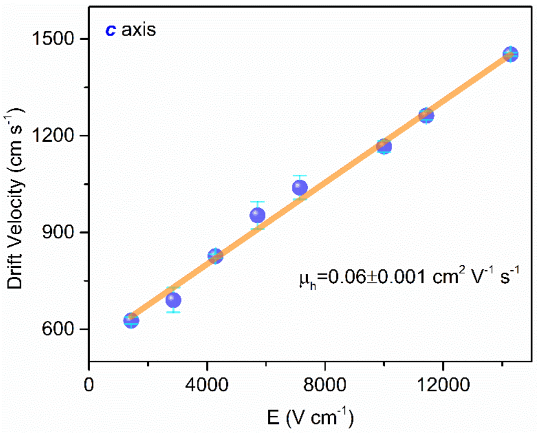

Fig. S15 Charge carrier transport behaviour in 4HPA single crystal along the *c* axis. Holes drift velocity changes with applied electric field and hole mobilities fitting ($cm^2 \cdot V^{-1} \cdot s^{-1}$)

Table. S1 Anisotropic charge mobility in 4HPA single crystal ($cm^2 \cdot V^{-1} \cdot s^{-1}$)

	a axis	*c* axis
Electron	4.17±0.040	2.60±0.05
Hole	N/A	0.06±0.001

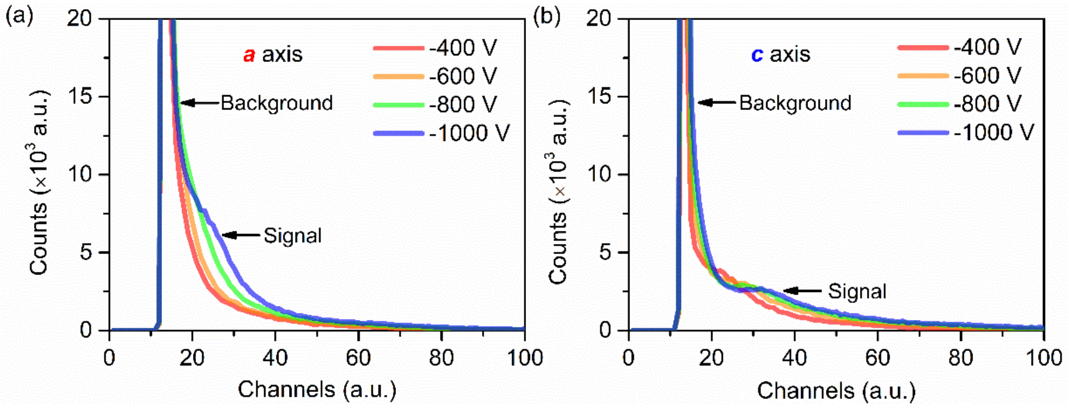

Fig. S16 Anisotropic charged particles spectra obtained by hole-only signals in 4HPA detectors. (a) Spectra at a series of bias voltages along the *a* axis, (b) Spectra at a series of bias voltages along the *c* axis. Compared with electron-only signal in Fig. 5, hole-only charged particles spectra show poorer detection ability for full energy peak.

SI 2 Drift velocity calculations

According to Fig. 3c, when the electric field at 4000 V cm^{-1}, the average drift time of electrons along the a axis is 0.5 μs, while the drift length is 0.25 cm, then the drift velocity can be calculated as 5×10^5 cm s^{-1}.

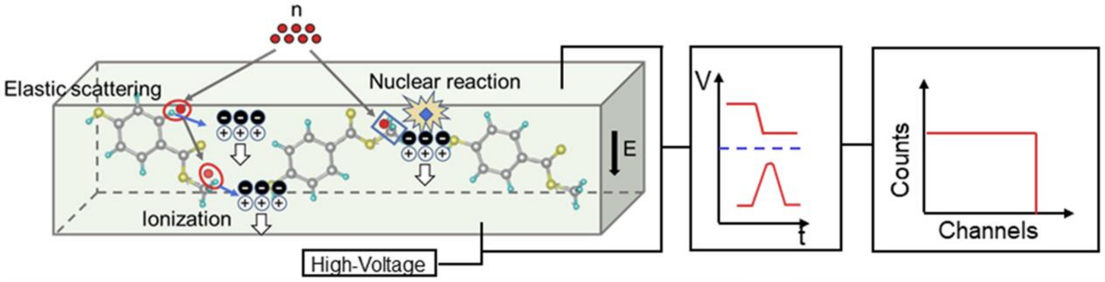

Fig. S17 Schematic diagram of direct fast neutron detection by 4HPA OSCS[4]. The direct detection of fast neutrons by 4HPA OSCS is due to 4HPA possesses high-density of H atoms (~2×10^{22} n cm^{-3}) that have largest interaction possibility with fast neutrons to produce charged particles, and at the same time, 4HPA also can detect charged particles as a semiconductor detector, therefore achieving direct detection of fast neutrons (Fig. S17), as we described in Ref[4]. It worth note that organic detectors like 4HPA OSCS has very high detection efficiency for direct fast neutron detection while most of inorganic semiconductors are not sensitive to fast neutrons.

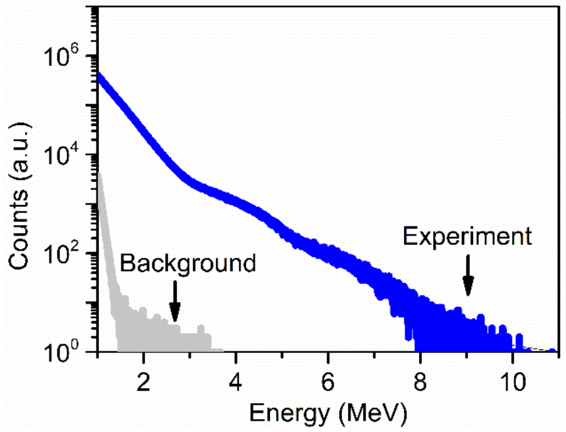

Fig. S18 Fast neutrons spectra measured by 4HPA sensors. For the neutron spectrum collection, the detection method is same with our previous work[4]. The 4HPA detector was positioned inside a copper chamber within a Pb room build by Pb bricks at a distance of 5 cm from the ^{241}Am-Be neutron source. The detector was powered by a high voltage power supply (ORTEC, 556), then the output signal was sent to pre-amplifier (ORTEC, 142PC) and amplifier (ORTEC, 572A) for signal amplification. The amplified signal was input to a digital analysis for data processing with a neutron spectrum output. For the neutron spectrum, the applied bias voltage is 700 V for a 4HPA detector with the thickness of 1mm, and the collection time is 30 min for both background and the neutron spectra.

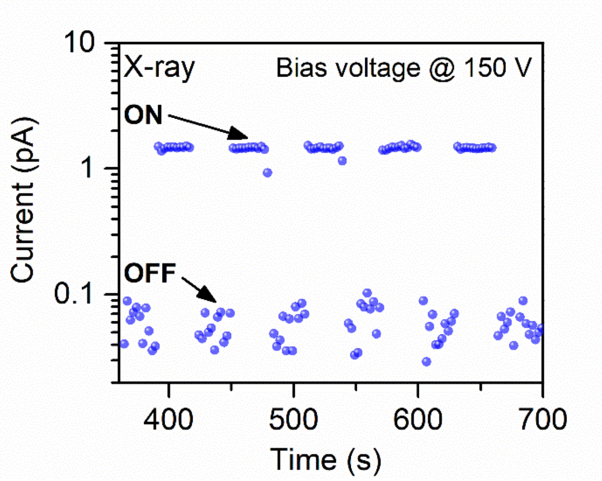

Fig. S19 Dark current fluctuation in 4HPA detectors with bias voltage of 150 V. 4HPA detectors also show very low dark current below 0.1 pA at the bias voltage of 150 V, resulting in extremely low dark current drift ~ 10^{-9} nA cm^{-1} s^{-1} V^{-1}. The low dark current drift and high S/J_{Dark} value indicate the highly stable X-ray response and high signal-to-noise ratio of 4HPA OSCS.

SI 3 Detection of Limit (LoD) of 4HPA detectors

Fig. S20 shows the typical *I-t* curves and corresponding calculated Signal-to-Noise Ratio (SNR) values of the 4HPA detectors (Fig. S21(a)). Although 0.35 μGy_{air} s^{-1} is the lowest dose rate in our measurement system, we estimated the minimum dose rate according to the SNR value larger than 3, which is around 0.02 μGy_{air} s^{-1} (20 nGy_{air} s^{-1}), as shown in Fig. S21(b). The LoD for 4HPA is lower than reported value for 4HCB with 0.29 μGy_{air} s^{-1}. We compared the LoD and S/J_{Dark} of 4HPA, 4HCB and other halide perovskite single crystalline detectors (Fig. S21(c))[5-13]. Although organic semiconductors normally have weaker X-ray absorption efficiency and lower charge mobility than inorganic single crystalline semiconductors, we achieved the comparable X-ray detection performances with halide perovskites possessing benefits of high atomic number (Z) and high charge mobility. In addition, compared with halide perovskite, 4HPA organic detectors also have better tissue-equivalent, biocompatible, flexible and low-cost properties, which make them promise for next-generation lightweight and human-friendly wearable or implantable X-ray dosimeters.

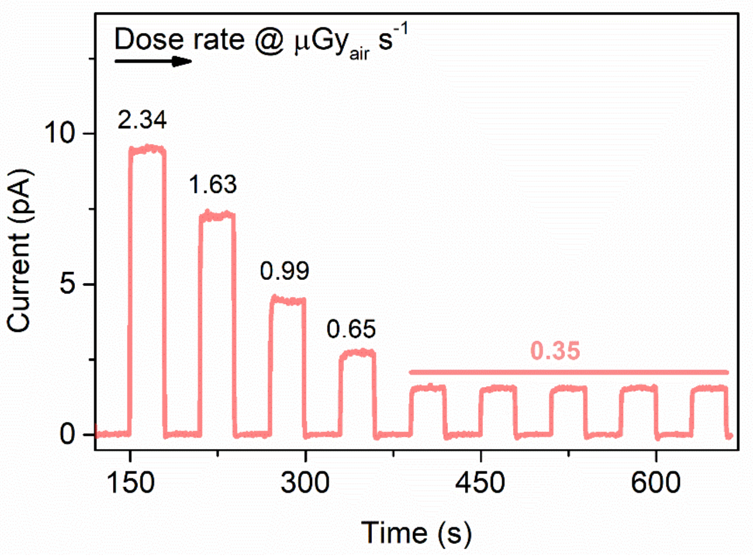

Fig. S20 X-ray detection photocurrent of 4HPA single crystal detectors changes with incident dose rate, bias voltage is 50 V.

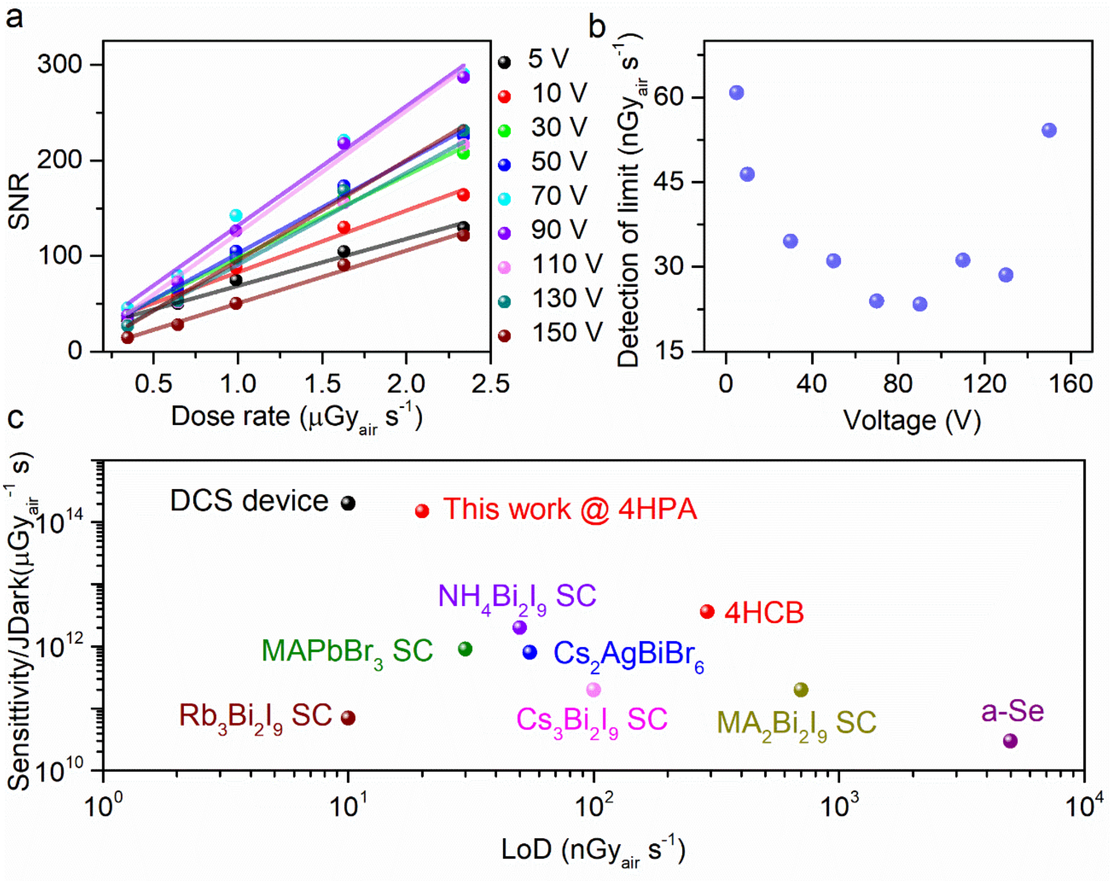

Fig. S21 X-ray LoD of 4HPA detectors. (a) SNR values changes with incident dose rate, (b) LoD values changes with bias voltage s of the 4HPA detectors, (c) comparison of 4HPA detectors in this paper and other X-ray detectors of organic and perovskite single crystal devices in term of the S/J_{Dark} and LoD values.

SI 4 X-ray Attenuation Efficiency (ε) of 4HPA Detector

For the X-ray beam with single photon energy, the attenuation efficiency[7],

$$\varepsilon(E) = 1 - \frac{I}{I_0} = 1 - e^{-\mu(E)\times\rho\times x} \qquad \text{(S3)}$$

where I is the transmitted intensity, I_0 the incident intensity, $\mu(E)$ is the total attenuation mass coefficient from XCOM: Photon Cross Sections Database[14] (Fig. S22(a)), ρ is the mass density of 4HPA detector and x is the thickness of the detector. As shown in Fig. S22(b), 2 mm-thick 4HPA detectors possess 100% absorption rate for X-rays below 10 keV, and the same attenuation efficiency of human tissue that indicates the very good tissue equivalence compared with other detectors with high atomic numbers like Si and CdTe.

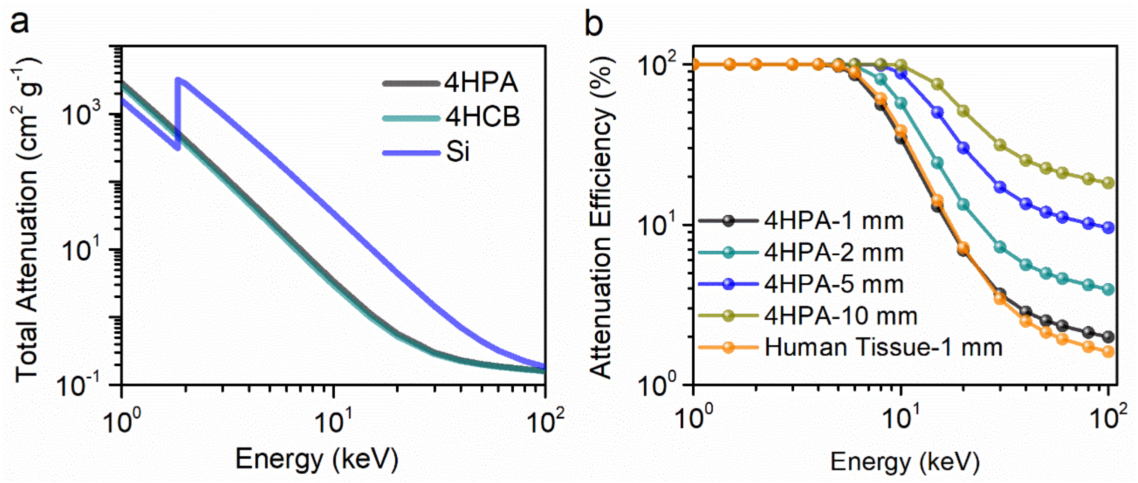

Fig. S22 X-ray absorption of 4HPA single crystal. (a) Total attenuation cross section comparison 4HPA, 4HCB, and Si, (b) X-ray attenuation efficiency of 4HPA detectors with series of thickness changes with incident photon energy.

SI 5 Charge Collection Efficiency and Detection Sensitivity of 4HPA Detectors

For 4HPA organic X-ray detectors, the Charge Collection Efficiency (CCE) was calculated by[15]:

$$CCE = \frac{I_R}{I_P} \tag{S4}$$

where I_R and I_P are measured and theoretical photocurrent under X-ray irradiation. I_P is defined as $I_P = \varphi\beta e$, where φ is the photo absorption rate, β is the maximum number of photo generated carriers per photon, and e is the elementary charge. The photo absorption rate is given by $\varphi = \varepsilon D m_s / E_{ph}$ photons s^{-1}, where ε is the attenuation efficiency, D is the dose rate, m_s is the sample mass, E_{ph} is the energy per photon[15].

In particular, β, the maximum number of carriers generated by per photon, which is estimated as $\beta = 2 \times \frac{E_{ph}}{\Delta E}$, ΔE is the electron-hole creation energy, is estimated as 3 times band gap (E_g=3.8 eV) of 4HPA[16], (around 175 electrons and holes generated by 1 keV photon).

Then, we calculated and performed theoretical and experimental X-ray photocurrent induced by 4HPA detectors, respectively. The tube voltage of X-ray is 40 kVp and the energy of X-ray photons in range of 0 ~ 40 keV. The estimated energy distribution of X-ray beam by using the software (SPEKTR 3.0), as shown in Fig. S23. SPEKTR 3.0 is kindly shared by the I-STAR lab at John-Hopkins University and can be freely downloaded from http://istar.jhu.edu/downloads/.

For theoretical photocurrent measurement, we need to calculate the equivalent absorption efficiency ($\overline{\epsilon}$) because the 4HPA detector has different attenuation efficiency ($\varepsilon(E)$) between photons with different energy. First, the distribution of photons energy ($N(E)$, red area) and the corresponded absorption rate ($\varepsilon(E)$, blue dots) are shown in Fig. S24(a), where the red filled area is the total energy of incident 40 kVp X-ray beam. Then, the total absorbed X-ray by the 2 mm-thick 4HPA detector is equal to

$$\int_{10}^{40} E \times \varepsilon(E) dE \tag{S5}$$

as shown in Fig. S24(b) (red area). Finally, the equivalent fraction of absorbed photons ($\bar{\epsilon}$) for 40 kVp X-ray beam in 4HPA detectors is calculated[17],

$$\bar{\varepsilon} = \frac{Total\ absorbed\ energy}{Total\ incident\ X\!-\!ray\ energy} \tag{S6}$$

which is around 10% for a 4HPA single crystals of 2 mm thickness under 40 kVp X-ray irradiation.

Then, we measured the Photocurrent-Time (I_R-t) curves of 4HPA detector (Size: 0.003 cm^3) with X-ray switching "ON/OFF" and decreased dose rate of 0.35 ~ 2.34 $\mu Gy_{air}\ s^{-1}$ under a bias voltage of 5~130 V (Fig. S25(a)). The I_P values corresponding to the measurement dose rate were also calculated, with compared with I_R (Fig. S25(b)). Then, the CCE values were calculated by I_R/I_P, which is up to 15000% at 150 V of bias voltage (Fig. S25(c)). The CCE larger than 100% normally due to the charge injection from electrodes, which may originate from the layer edge states on 4HPA surface. Due to the high CCE values, the 4HPA detectors can achieve high Sensitivity of 330 $\mu C\ Gy_{air}\ cm^{-2}$ or 16612 $\mu C\ Gy_{abs}^{-1}\ cm^{-3}$ (Fig. S25(d-e)) for 40 kVp X-ray detections. Then, the X-ray detection Sensitivity (S)/Dark current density (J_{Dark}) was calculated, which was as high as 1.5×10^{14} μGy_{air}^{-1} s (Fig. S25(f))[13].

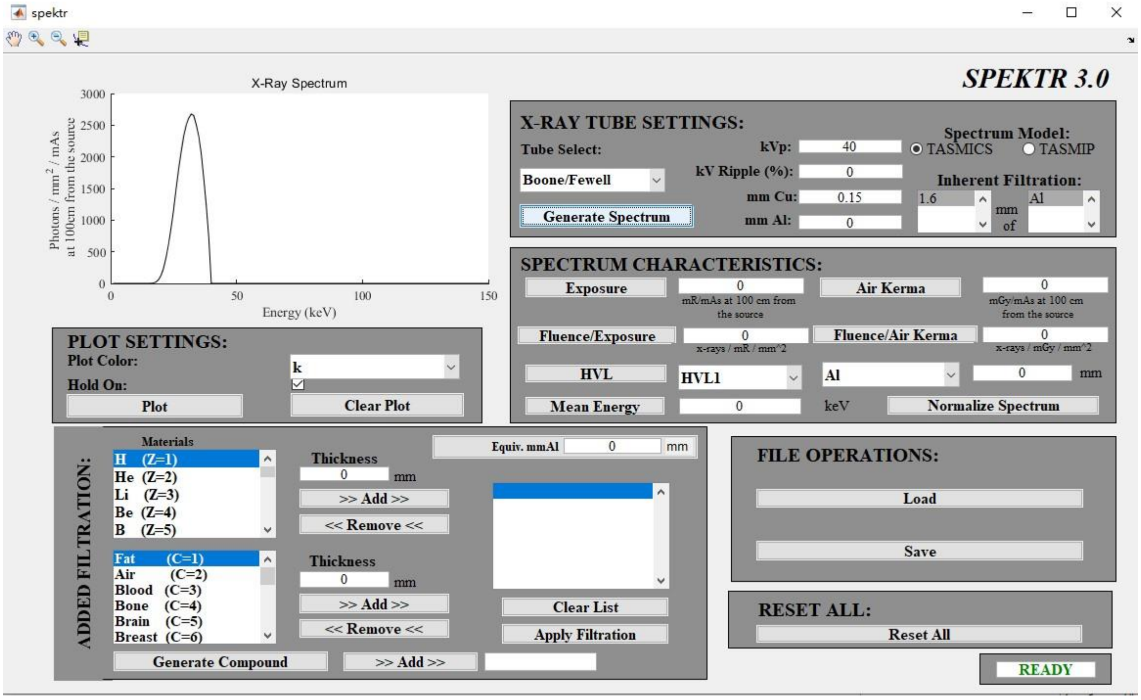

Fig. S23 Incident X-ray energy spectrum simulated by SPEKTR.

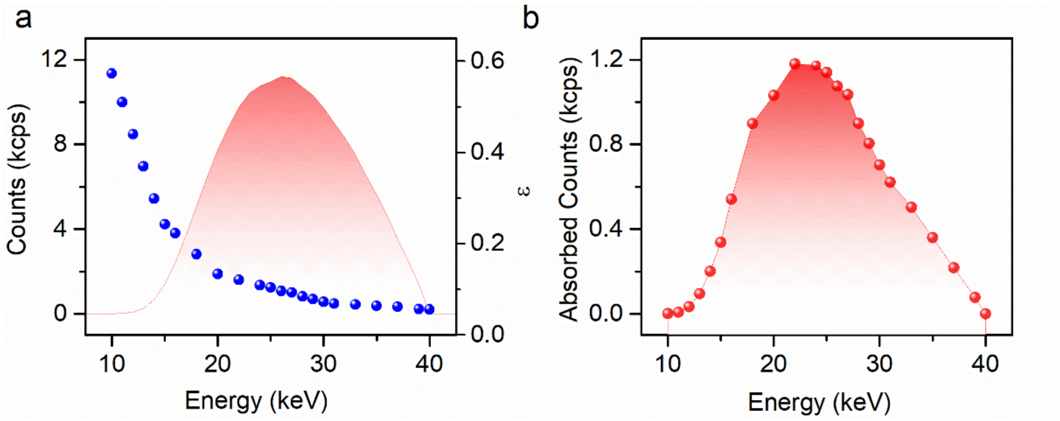

Fig. S24 Effective attenuation efficiency (ε) of 4HPA detectors for 40 kVp X-rays. Incident X-ray energy spectrum simulated by SPEKTR (a) Energy distribution (red area) and fraction of absorption (Blue dots) of 40 kVp X-ray photons, (b) total absorbed X-ray energy by 4HPA single crystal under 40 kVp X-ray irradiation.

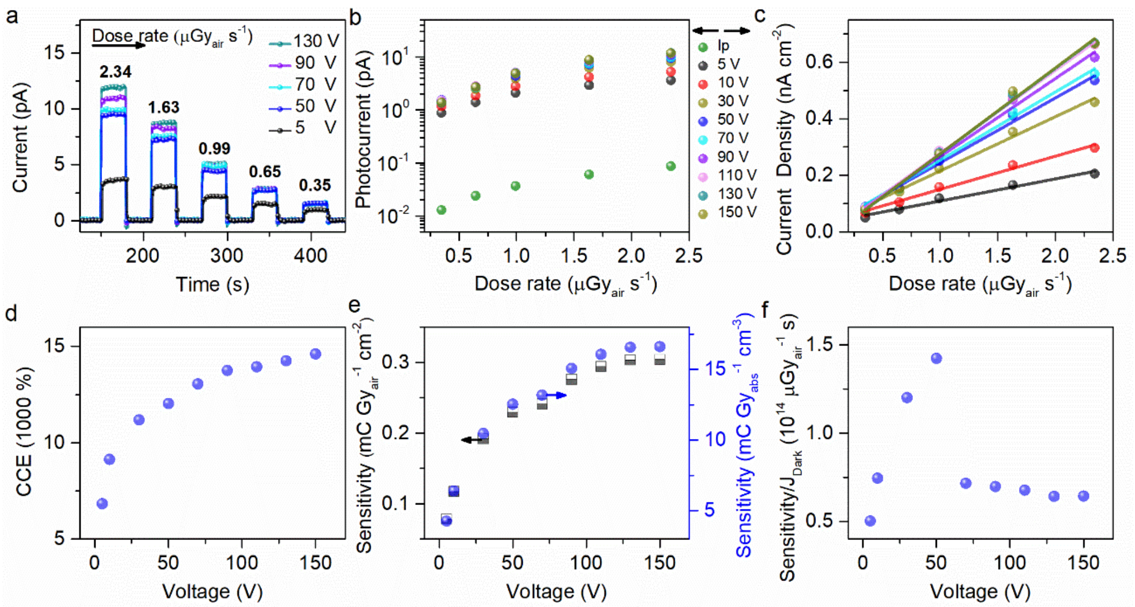

Fig. S25 X-ray detection response of 4HPA single crystal detectors. (a) *I*-*t* curve with reduced incident X-ray dose rate, (b) I_R and I_P changes with incident X-ray dose rate, (c) calculated sensitivity of 4HPA detectors, bias-voltage-dependent (d) Charge Collection Efficiency (CCE) and (e) the detection sensitivity, and (f) the ratio of detection sensitivity and dark current density (Sensitivity/J_{Dark}). The effective detector size is 0.003 cm^3, thickness is 0.19 cm, bias voltage is 50 V. The Tube current of X-rays is 40 kV, tube current changes from 5~60 μA, 0.15 mm Cu is used as attenuator.

SI 6 Degradation with radiations

At first, we evaluated the long-time *I-t* curves with "ON/OFF" switching behaviors of 4HPA detectors. With the increasing X-ray dose rate, as shown in Fig. S26(a), after 50 cycles (continuous operation of 3100 s), the 4HPA detectors didn't show any degradation. Then, we measured the 100 "ON/OFF" switching cycles (continuous operation of 6000 s) under constant X-ray irradiation with dose rate of 19.18 mGy_{air} s^{-1} and bias voltage of 150 V. No degradation happens in both dark and photocurrent (Fig. S26(b)).

In addition, long-time current drift stability measurements of 4HPA devices were also carried out with continuous 2-hour dark current measurement and photocurrent measurement with X-ray irradiation (dose rate of 19.18 mGy_{air} s^{-1}, total dose of 138.096 Gy_{air}). The result shows both the dark current drift (around 10^{-9} nA cm^{-1} s^{-1} V^{-1}) and photocurrent drift are very small, and no degradation occurs after continuous 2-hour device work period (Fig. S27).

In further, we utilized 150 kVp X-ray (dose rate of 19.18 mGy_{air} s^{-1}) to irradiate 4HPA detectors for 10 hours, with total irradiation dose of 690.48 Gy_{air} (upper limit of our X-ray generator). We compared the photodetection properties of before and after 10-hour irradiation, as shown in Fig. S28. Both dark current and photocurrent didn't show any obvious degradation.

These results indicate 4HPA detectors show very good radiation stability and long-term work stability at high electric field.

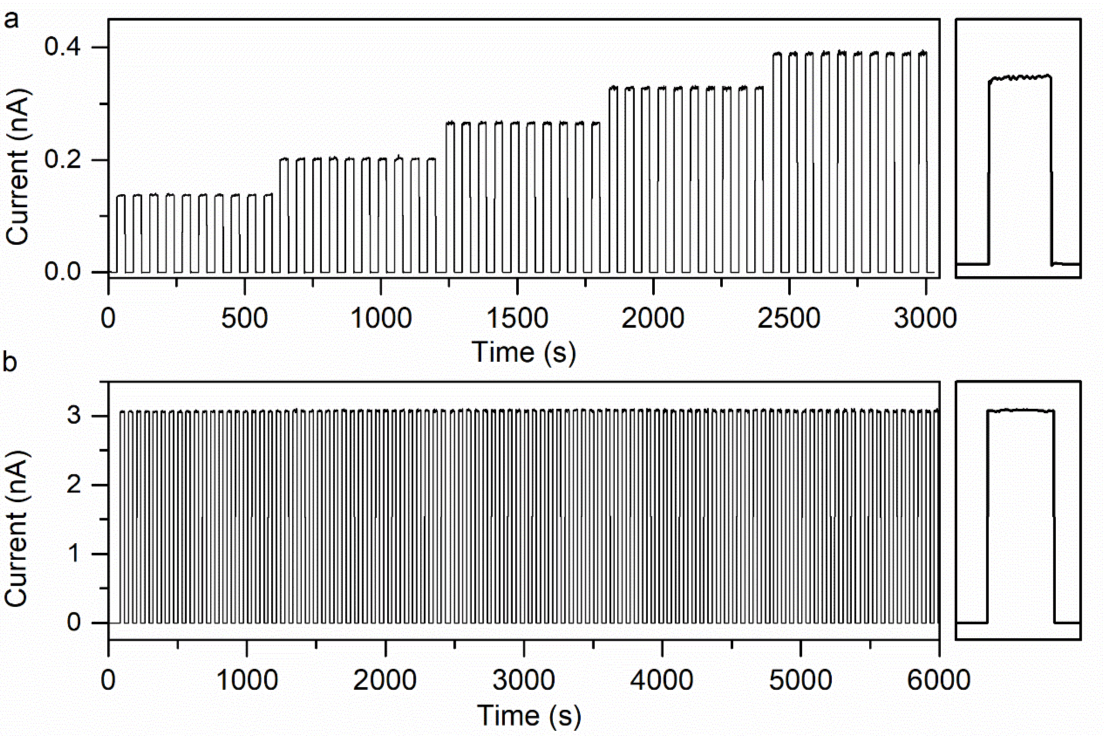

Fig. S26 X-ray detection long-term work stability. (a) X-ray photocurrent I-T cycles change with increased dose rate, (b) X-ray photocurrent I-T cycles when the work time continues to 6000 s with bias voltage of 150 V.

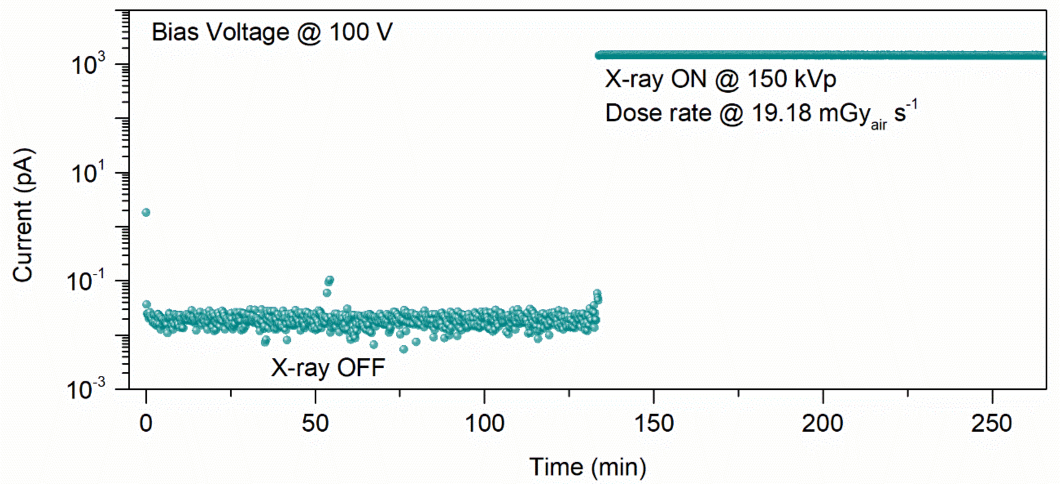

Fig. S27 X-ray detection long-term work stability of 4HPA detectors with continue 120 min work for X-ray detection.

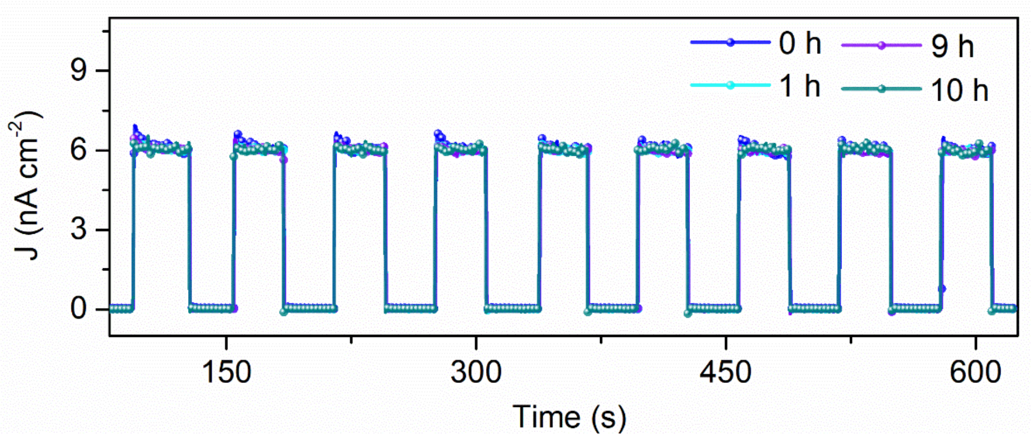

Fig. S28 X-ray detection long-term work stability after continuously detection of 0 h, 9 h, 1 h, and 10 h and 150 kVp X-ray dose rate of 19180 μGy_{air} s^{-1}, total irradiation dose of 690.48 $Gy_{air.}$

Table. S2. Advantages/disadvantages of current dosimeters and advances of biocompatible 4HPA organic detectors

Materials	Advantages	Disadvantages	Advance of 4HPA detectors
**Thermoluminescent dosimeter (TLD)**	• Small size<br>• Cheap<br>• Available in various forms	• Not real-time<br>• Need complex calibration	• Real-time<br>• Energy-resolved<br>• Tissue equivalent
**Ion chamber**	• Real-time<br>• Precise	• Bulky size and visible<br>• High voltage supply	• Compact & small volume<br>• Low-voltage supply (thick film)
**Si**	• High carrier mobility<br>• High energy resolution<br>• Compact<br>• High spatial resolution<br>• Fast response	• Low resistivity<br>• Poor detection limit (~mGy)<br>• T-dependent response<br>• No fast neutron detection<br>• Non-tissue equivalent<br>• Limited biocompatibility<br>• Large density (2.33 g cm^{-3})<br>• Brittle thin-film wafer<br>• High-cost melt fabrication	• High resistivity (10^{12} Ω cm)<br>• Superior detection limit (20 nGy)<br>• Insensitive to T and visible light<br>• Direct fast neutron detection<br>• Tissue-equivalent<br>• Superior biocompatibility<br>• Light weight (1.25 g cm^{-3})<br>• Flexibility<br>• Low-cost solution method

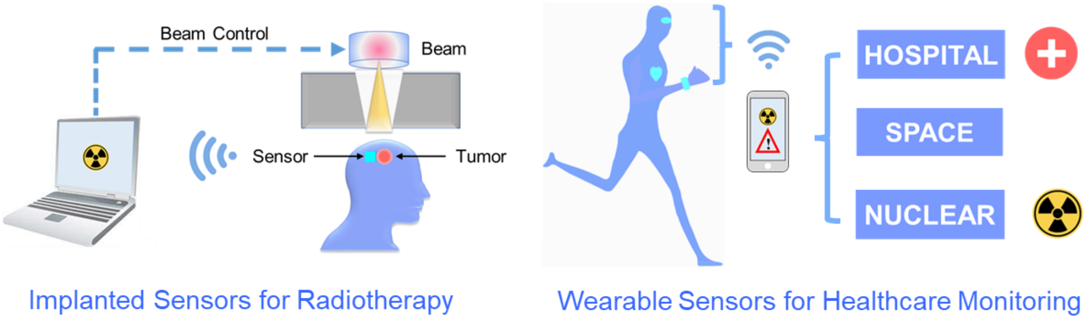

Fig. S29 Potential application of biocompatible and fast-response 4HPA OSCS. 4HPA OSCS is promising as wearable/implanted sensors for in-vivo and real-time healthcare monitoring for tumor therapy and radiation safety control.

References

1 Zhao, D. *et al.* Purely organic 4HCB single crystals exhibiting high hole mobility for direct detection of ultralow-dose X-radiation. *J. Mater. Chem. A* **8**, 5217-5226, doi:10.1039/c9ta12817d (2020).

2 Jubu, P., Yam, F., Igba, V. & Beh, K. Tauc-plot scale and extrapolation effect on bandgap estimation from UV–vis–NIR data–a case study of β-Ga_2O_3. *J. Solid State Chem.* **290**, 121576 (2020).

3 Brown, F. C. Temperature dependence of electron mobility in AgCl. *Phys. Rev.* **97**, 355 (1955).

4 Zhao, D. *et al.* Direct Detection of Fast Neutrons by Organic Semiconducting Single Crystal Detectors. *Adv. Funct. Mater.* **32**, 2108857, doi:10.1002/adfm.202108857 (2022).

5 Wei, W. *et al.* Monolithic integration of hybrid perovskite single crystals with heterogenous substrate for highly sensitive X-ray imaging. *Nat. Photonics.* **11**, 315-321 (2017).

6 Zhuang, R. *et al.* Highly sensitive X-ray detector made of layered perovskite-like $(NH_4)_3Bi_2I_9$ single crystal with anisotropic response. *Nat. Photonics.* **13**, 602-608 (2019).

7 Pan, W. *et al.* $Cs_2AgBiBr_6$ single-crystal X-ray detectors with a low detection limit. *Nat. Photonics.* **11**, 726-732 (2017).

8 Zhang, Y. *et al.* Nucleation-controlled growth of superior lead-free perovskite $Cs_3Bi_2I_9$ single-crystals for high-performance X-ray detection. *Nat. Commun.* **11**, 2304 (2020).

9 Liu, Y. *et al.* Inch-size 0D-structured lead-free perovskite single crystals for highly sensitive stable X-ray imaging. *Matter* **3**, 180-196 (2020).

10 Xia, M. *et al.* Unveiling the structural descriptor of $A_3B_2X_9$ perovskite derivatives toward X-ray detectors with low detection limit and high stability. *Adv. Funct. Mater.* **30**, 1910648 (2020).

11 Kasap, S. *et al.* Amorphous selenium and its alloys from early xeroradiography to high resolution X-ray image detectors and ultrasensitive imaging tubes. *Physica Status Solidi B* **246**, 1794-1805 (2009).

12 Matt, G. J. *et al.* Sensitive direct converting X-ray detectors utilizing crystalline $CsPbBr_3$ perovskite films fabricated via scalable melt processing. *Adv. Mater. Interfaces* **7**, 1901575 (2020).

13 Jin, P. *et al.* Realizing nearly-zero dark current and ultrahigh signal-to-noise ratio perovskite X-ray detector and image array by dark-current-shunting strategy. *Nat. Commun.* **14**, doi:10.1038/s41467-023-36313-6 (2023).

14 Berger, M. J. e. a. XCOM: Photon Cross Sections Database: NIST Standard Reference Database 8 (NIST, 2013). https://www.nist.gov/pml/xcom-photon-cross-sections-database.

15 Alig, R. C. & Bloom, S. Electron-Hole-Pair Creation Energies in Semiconductors. *Phys. Rev. Lett.* **35**, 1522-1525, doi:10.1103/PhysRevLett.35.1522 (1975).

16 Zhao, D. *et al.* Photoconductive gain under low-flux X-ray irradiation in 4HCB organic single crystal detectors. *Appl. Phys. Express.* **13**, 071004, doi:10.35848/1882-0786/ab9adb (2020).
